# Supplementary figures and images for: Monitoring newt communities in urban area using eDNA metabarcoding
Source: PeerJ. 2021 Nov 26;9:e12357. doi: 10.7717/peerj.12357 (PMC8628619; doi:10.7717/peerj.12357)

# Map of Europe

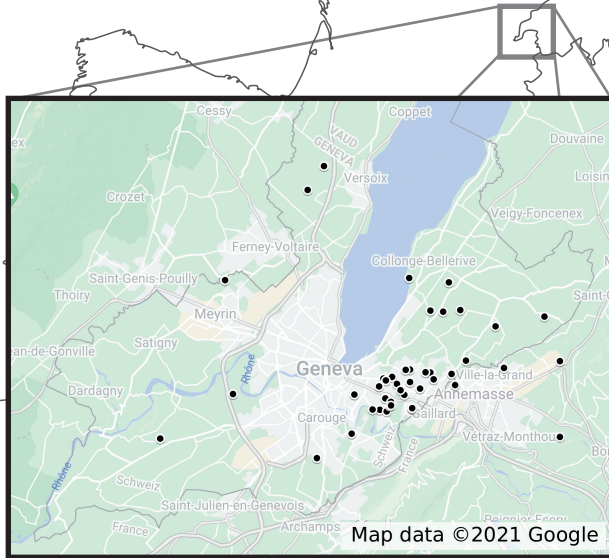

**Area of study**  
**Geneva, Switzerland**

Supplement: Supplemental Information 2 [file peerj-09-12357-s002.pdf]
