# Supplementary material for: Monitoring newt communities in urban area using eDNA metabarcoding
Source: PeerJ. 2021 Nov 26;9:e12357. doi: 10.7717/peerj.12357 (PMC8628619; doi:10.7717/peerj.12357)
Supplement: Supplemental Information 1 [file peerj-09-12357-s001.pdf]

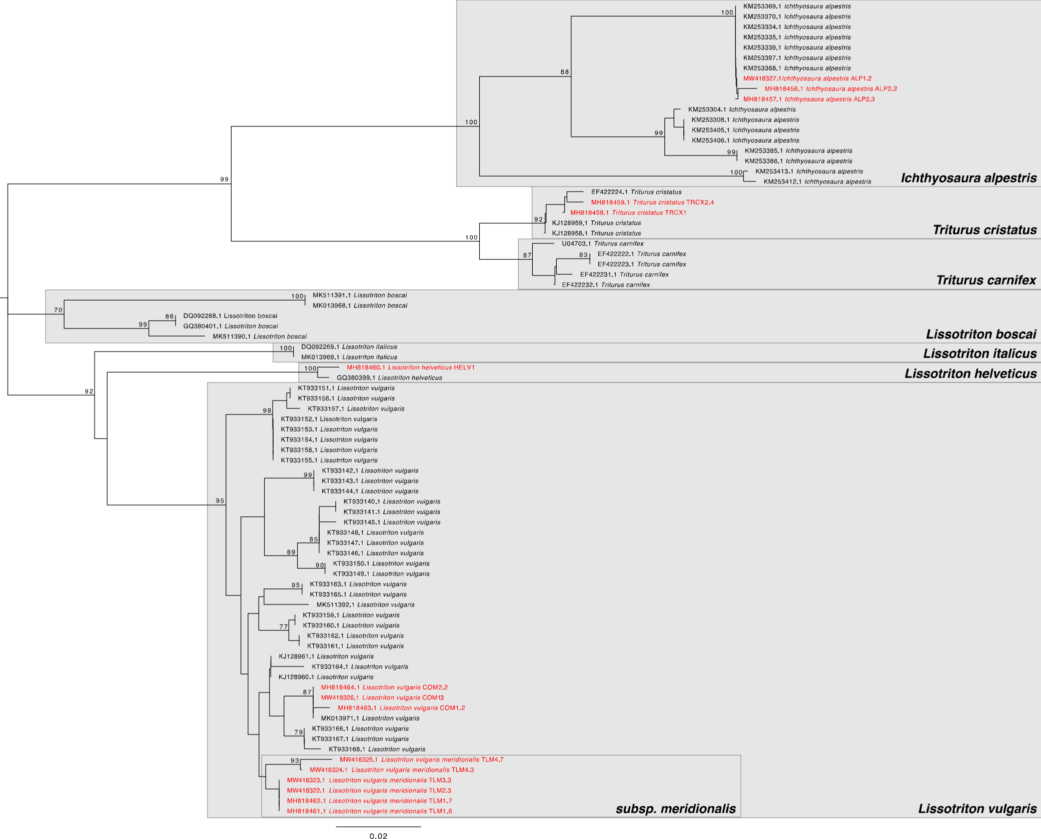

KM253369.1 Ichthyosaura alpestris
KM253370.1 Ichthyosaura alpestris
KM253334.1 Ichthyosaura alpestris
KM253335.1 Ichthyosaura alpestris
KM253339.1 Ichthyosaura alpestris
KM253397.1 Ichthyosaura alpestris
KM253368.1 Ichthyosaura alpestris
MW418327.1 Ichthyosaura alpestris ALP1.2
MH818456.1 Ichthyosaura alpestris ALP2.2
MH818457.1 Ichthyosaura alpestris ALP2.3
KM253304.1 Ichthyosaura alpestris
KM253308.1 Ichthyosaura alpestris
KM253405.1 Ichthyosaura alpestris
KM253406.1 Ichthyosaura alpestris
KM253385.1 Ichthyosaura alpestris
KM253386.1 Ichthyosaura alpestris
KM253413.1 Ichthyosaura alpestris
KM253412.1 Ichthyosaura alpestris
Ichthyosaura alpestris
EF422224.1 Triturus cristatus
MH818459.1 Triturus cristatus TRCX2.4
MH818458.1 Triturus cristatus TRCX1
KJ128959.1 Triturus cristatus
KJ128958.1 Triturus cristatus
Triturus cristatus
U04703.1 Triturus carnifex
EF422222.1 Triturus carnifex
EF422223.1 Triturus carnifex
EF422231.1 Triturus carnifex
EF422232.1 Triturus carnifex
Triturus carnifex
MK511391.1 Lissotriton boscai
MK013968.1 Lissotriton boscai
DQ092268.1 Lissotriton boscai
GQ380401.1 Lissotriton boscai
MK511390.1 Lissotriton boscai
Lissotriton boscai
DQ092269.1 Lissotriton italicus
MK013969.1 Lissotriton italicus
Lissotriton italicus
MH818460.1 Lissotriton helveticus HELV1
GQ380399.1 Lissotriton helveticus
Lissotriton helveticus
KT933151.1 Lissotriton vulgaris
KT933156.1 Lissotriton vulgaris
KT933157.1 Lissotriton vulgaris
KT933152.1 Lissotriton vulgaris
KT933153.1 Lissotriton vulgaris
KT933154.1 Lissotriton vulgaris
KT933158.1 Lissotriton vulgaris
KT933155.1 Lissotriton vulgaris
KT933142.1 Lissotriton vulgaris
KT933143.1 Lissotriton vulgaris
KT933144.1 Lissotriton vulgaris
KT933140.1 Lissotriton vulgaris
KT933141.1 Lissotriton vulgaris
KT933145.1 Lissotriton vulgaris
KT933148.1 Lissotriton vulgaris
KT933147.1 Lissotriton vulgaris
KT933146.1 Lissotriton vulgaris
KT933150.1 Lissotriton vulgaris
KT933149.1 Lissotriton vulgaris
KT933163.1 Lissotriton vulgaris
KT933165.1 Lissotriton vulgaris
MK511392.1 Lissotriton vulgaris
KT933159.1 Lissotriton vulgaris
KT933160.1 Lissotriton vulgaris
KT933162.1 Lissotriton vulgaris
KT933161.1 Lissotriton vulgaris
KJ128961.1 Lissotriton vulgaris
KT933164.1 Lissotriton vulgaris
KJ128960.1 Lissotriton vulgaris
MH818464.1 Lissotriton vulgaris COM2.2
MW418326.1 Lissotriton vulgaris COM13
MH818463.1 Lissotriton vulgaris COM1.2
MK013971.1 Lissotriton vulgaris
KT933166.1 Lissotriton vulgaris
KT933167.1 Lissotriton vulgaris
KT933168.1 Lissotriton vulgaris
MW418325.1 Lissotriton vulgaris meridionalis TLM4.7
MW418324.1 Lissotriton vulgaris meridionalis TLM4.3
MW418323.1 Lissotriton vulgaris meridionalis TLM3.3
MW418322.1 Lissotriton vulgaris meridionalis TLM2.3
MH818462.1 Lissotriton vulgaris meridionalis TLM1.7
MH818461.1 Lissotriton vulgaris meridionalis TLM1.6
subsp. meridionalis
Lissotriton vulgaris
0.02
